# The New Discovery about Pneumonia

**Published:** 1916-01

**Authors:** Leonard Keene Hirshberg

**Affiliations:** Johns Hopkins


### The New Discovery About Pneumonia.

BY DR. LEONARD KEENE HIRSHBERG, A. B., M. A., M. D., JOHNS HOPKINS.

When Professor Paul Ehrlich announced four years ago that the loathsome blood disease which had required a slow and tedious course of treatment by mercury could be dramatically cured by one or two hypodermic injections of a newly discovered drug the whole medical world sat up and began to take notice. The fervor of investigation which had spent fifteen years of fury in unearthing vaccines, serums and antitoxins was given pause, and the very savants such as Sir Almoth E. Wright, who had extended the application of anti-typhoid vaccine, began again to turn their distracted attention to drugs or "chemotherapy" as disease exterminators.

It was truly, however, more the anarchistic skepticism of Sir William Osler about the efficacy of drugs in curing diseases, more than the prominence of anti-toxins and vaccines, that had influenced medical men to scoff at medicines of a chemical nature. The peans of praise bestowed upon drugs and herbs, chemicals and potions by doctors of previous generations, as well as by patent medicine exploiters and advertising pharmacies, were as wide of the mark, so far from the truth that Dr. Osler said in his nihilistic manner that the only drugs that were worth an oyster-shucker's oath were those that smelled good, tasted good, looked good and were at the same time harmless.

His successor, Dr. Lewellyn F. Barker, taught that there are but six efficacious drugs known, towit: mercury, iron, quinine, salicylic acid and two more. Professor William Halsted, the noted Johns Hopkins surgeon, is even more radical. He says that if he were cast away upon a Pacific Island, and compelled to practice his profession among savages, he would choose a hatful of calomel pills as his complete equipment.

Be all this as it may, times are certainly changing from the medical points of view. Knocked into a cocked hat by the iconoclastic blows of serum-therapy, professional doubt, Oslerismic nihilism, Christian Science, New Thought, Fletcherism and other personal and impersonal fads, chemical therapy under the impetus of scientific tests and laboratory control bids fair to soon more than come into its own. For not only have the skillful and conservative Professors Ehrlich and Neisser of Germany come to its support, but no less a person than Sir Almot E. Wright of London.

Early in the year just closed Professor Morgenroth of Germany built up in his laboratory a new synthetic drug with the terrifying polysyllabic name ethylhydrocupreinhydrochlorate. Formidable as this eleven-syllable name would seem, it is so harmless to the human tissues in proper dilutions that wonderfully beneficial things are being done with it.

As soon as Professor Morgenroth announced his expectations with regard to ethylhydrocupreinhydrochlorate, Sir Almoth Wright became interested. Professor Morgenroth injected this new drug into animals and at once saw startling results. His experiments are destined to stand out as landmarks in the history of the new scientific discoveries in the healing powers of drugs. They furnish the anxious world for the first time with the certain proof that microscopic diseases such as pneumonia and meningitis, strictly bacterial diseases as distinct from protozoal and animalicule infections, can be both prevented and cured by the use of the correct chemical.

Dr. Morgenroth inoculated mice and rats with the bacteria that are responsible for pneumonia. This pneumococcus is always fatal to rodents; so fatal that when given an infinitesimal number of the germs they die with 100 per cent fatalities. The German physician divided his rodents into three groups of, let us say, one hundred each. Into the first hundred he injected a very weak solution of the drug ethylhydrocupreinhydrochlorate, into the second group he injected at once the pneumococci, and then he inoculated the third hundred with the pneumococci.

Now he returned to the first hundred which had already received the drug and gave them a dose of germs large enough to kill a mastodon. Three or four hours later he gave the third division each a dose of the drug. Meanwhile, you see, the second division of one hundred mice were the only ones that had not been given the medicine. What was the result? Why, the most startling indeed. For the hundred mice that had first received the drug only later to be given the usually fatal malady all survived. The drugs prevented any development of the ailment. While the third phalanx which had first developed symptoms before the drug was injected was also entirely well. The drug had cured these. But not a mother's son lived in the second hundred animals. Unhappily for them, Professor Morgenroth omitted to give them any ethylhydrocupreinhydrochlorate.

You might naturally ask, "What have ailments of these filthy creatures to do with human pneumonia?" To which answer may be made that well laid plans hold or "gangaglee" for both mice and men. Goose sauce may not be exactly the same as gander sauce, but sauce is sauce. Professor Morgenroth made no statement whatsoever about pneumonia when he said the new drug prevented and cured mice with an infection from the pneumonia parasite. That is where Sir Almoth E. Wright steps into the breach.

With his practical British vision, Sir Almoth at once recognized the great value his Teutonic colleague's observations would have for mankind, if his experiments could be extended and successfully applied to the broader problem of pneumococcus infections in man. With this idea in view, with the hope of evolving the boon towards which medical men have aimed since the golden days of Pericles, Sir Almoth now announces the successful preliminary observations made by him in the London hospitals.

With the idea then of ridding the human race of the eternal scourge—pneumonia—he began his experiments on healthy men, because it is to the advantage of the sick that some sort of guide or standard be found for the drug, and because men in the vigor of health and strength may be expected to give timely warning of all sensations and impressions received during the treatment. Then, after this was determined, upon patients who had fallen ill with pneumonia.

It is certainly not desirable to state the dose of the new synthetic preparation that Professor Wright found his healthy men could stand, and which turned out afterwards to prove of such great service for his pneumonia patients. For it is too early to say that it may not be necessary to increase or decrease the amount. However that may be, this is the first authentic announcement from Sir Almoth himself that ethylhydrocupreinhydrochlorate does cure pneumonia.

The number of pneumonia patients treated by the British physician and his colleagues is rapidly growing. Since, however, only one year has elapsed and the number of pneumonia patients in one season even in so large a metropolis as London cannot be great enough to absolutely prove conclusions. Since, also, the pneumonia in one year is so much milder than other years that even the most dramatic series of cure may turn out to be more apparent than real, it will be wise for the usually impatient public to hold their horses long enough to have the new hailed drug tested on pneumonia patients of many climes over a period of several years before the new discovery is accepted as undoubtedly confirmed.

Unquestionably, however, this new drug and this new method is of vital import to man, and it is more than a remote hope that at last the savants of the medical laboratories have hit upon a cure of the marshal of the men of death, towit, pneumonia.

---

Macht concludes that alcohol after phenol poisoning not only does not act as an antidote but aggravates the symptoms and hastens death.—*Critic and Guide.*

---

### It's a Pretty Good Plan to Forget It.

If you see a tall fellow ahead of the crowd,
A leader of men, seaching fearless and proud,
And you know a tale whose mere telling aloud
Would cause his proud head to in anguish be bow'd,—
It's a pretty good plan to forget it!

If you know of a skeleton hidden away
In a closet, and guarded, and kept from the day
In the dark; whose showing, whose sudden display,
Would cause grief and sorrow, and lifelong dismay,—
It's a pretty good plan to forget it!

If you know of a spot in the life of a friend,
(We all have such spots concealed, world without end)
Whose touching, his heart-strings would play on, and rend,
Till the shame of its showing no grieving could mend,—
It's a pretty good plan to forget it!

If you know of a thing that will darken the joy
Of a man or woman, a girl or a boy;
That will wipe out a smile, or the least way annoy
A fellow; or cause any gladness to cloy,—
It's a pretty good plan to forget it!

If you know of a thing, just the least little sin,
Whose telling would cork up a laugh, or a grin,
Of a man you don't like—for the Lord's sake keep it in!
Don't, don't be a knocker; right here stick a pin!
It's a pretty good plan to forget it! —*Author unknown.*

